# Strigol induces germination of the facultative parasitic plant *Phtheirospermum japonicum* in the absence of nitrate ions

**DOI:** 10.1080/15592324.2022.2114647

**Published:** 2022-08-22

**Authors:** Satoshi Ogawa, Ken Shirasu

**Affiliations:** a RIKEN Center for Sustainable Resource Science, Yokohama, Kanagawa, Japan; b Graduate School of Science, the University of Tokyo, Tokyo, Japan

**Keywords:** parasitic plants, germination, strigolactones, nitrate, KAI2

## Abstract

Root parasitic plants in the family Orobanchaceae, such as *Striga* and *Orobanche* spp., infest major crops worldwide, leading to a multibillion-dollar loss annually. Host-derived strigolactones (SLs), recognized by a group of α/β hydrolase receptors (KAI2d) in these parasites, are important determinants for germinating root parasitic plants near the roots of host plants. *Phtheirospermum japonicum*, a facultative hemiparasitic Orobanchaceae plant, can germinate and grow in the presence or absence of the host and can also exhibit root chemotropism to host-derived SLs that are perceived via KAI2d. However, the importance of SLs in *P. japonicum* germination remains unclear. In this study, we found that germination of *P. japonicum* was suppressed in the absence of nitrate ions and that germination of *P. japonicum* was promoted by exogenous strigol, an SL, under such conditions. We propose a model in which *P. japonicum* may select either independent living or parasitism in response to ambient nitrogen conditions and host presence.

## Introduction

Parasitic plants in the family Orobanchaceae connect their roots to a wide range of plant species to derive nutrients and water.^1^ Infestation by such root parasites as *Striga* and *Orobanche* spp. are serious threats to global food security, causing annual losses of several billion dollars in crop production.^2,3^ These parasites produce a large number of small seeds that are viable for decades in soil and are, therefore, difficult to eliminate from fields once invaded.^4,5^ Hence, it is important to control parasite germination to suppress the infestation of host plants.

One of the key traits of obligate Orobanchaceae parasites, *i.e*., those that must have a host for survival, is host-stimulated germination. Seeds of obligate parasites such as *Striga* and *Orobanche* spp. have limited energy resources. To survive, obligate parasites must germinate near the host for immediate infection after germination. Accordingly, these parasites require host-derived stimulants for germination, in addition to water, oxygen, and a moderate temperature.^6^ Strigolactones (SLs), a group of terpenoids found in host-root exudates,^7^ are well-studied germination stimulants for such parasites.^8–10^

To recognize exogenous host-derived SLs, the Orobanchaceae parasites have evolved a group of α/β hydrolase receptors designated KAI2d, which originated from KARRIKIN INSENSITIVE 2 (KAI2)/HYPOSENSITIVE TO LIGHT (HTL) (hereafter KAI2).^11,12^ KAI2, which can respond to smoke-derived butenolide compounds called karrikins (KARs), are widely conserved in dicotyledonous plants and are required to activate signaling by recognizing unidentified endogenous KAI2 ligands (KLs).^13,14^ Most dicotyledonous plants have a single *KAI2* gene. By contrast, Orobanchaceae parasites have multiple *KAI2* genes that are categorized into three clades: a “conserved” type (*KAI2c*) that is similar to *KAI2* in other angiosperms and is found in most Lamiids, an “intermediate” type (*KAI2i*) found in Lamiales with a few exceptions such as *Orobanche* spp., and a “divergent” type (*KAI2d*) conserved only in Orobanchaceae parasites. Phylogenetic analyses showed that *KAI2d* genes were rapidly duplicated.^11,12,15^ Although KAI2d originated from KAI2, KAI2d recognize SLs but not KARs.^11,16^ Germination assays using the *Arabidopsis thaliana kai2* mutants revealed that KAI2 and KAI2d contribute to promoting KAR/KL-stimulated germination and SL-stimulated germination, respectively.^11,17^

The genome of the facultative hemiparasitic plant *Phtheirospermum japonicum* has seven *KAI2d* candidates.^18^ We recently discovered that at least two KAI2d proteins, designated PjKAI2d2 and PjKAI2d3.2, are important for root tropism to host-derived SLs in *P. japonicum*.^17^ However, the relationships between exogenous SLs and germination of *P. japonicum* remain unclear. We wondered if as a facultative parasite *P. japonicum* would germinate when the ambient nutrition was sufficient to survive alone and that in the case of low nutrient conditions, such as low nitrogen sources, the parasite would not germinate until ensuring that hosts are nearby to infect. In previous studies using *P. japonicum*, only nutrient-rich media were used for germination, and the effects of nutrient conditions on germination were not tested.^19–21^

Here, we show that *P. japonicum* seed germination was hindered in the absence of nitrate ions. In addition, we found that exogenous strigol, an SL, enhanced the germination of *P. japonicum* in nitrate-deficient conditions. In combination with our previous findings demonstrating that steps for host tropism are promoted by exogenous SLs and attenuated by ambient nitrogen sources,^17^ we propose a model for determining whether *P. japonicum* can live independently or become a parasite.

## Materials and methods

### Chemicals

Karrikin 1 (Catalog Number 025 7391), karrikin 2 (Cat. No. 025 6821) and *rac*-strigol (Cat. No. 025 6691) were purchased from Olchemim. These chemicals were stored away from moisture at −20°C as 10 mM stocks in dimethyl sulfoxide (DMSO) (Wako, Cat. No. 043–07216).

### Plant materials, growth conditions, and germination assays

*P. japonicum* (Thunb.) Kanitz seeds that were at least 1-month-old were used for all experiments. For germination assays, seeds were sterilized for 5 minutes with a 1:10 (v/v)-diluted commercial bleach solution (Kao, Tokyo, Japan), followed by at least 5 rinses with sterilized water. The sterilized seeds were sown on solid media without nutrients (0.7% (w/v) INA agar, pH 5.8) or solid media containing nutrients (0.8% (w/v) INA agar, pH 5.8). Concentrations of each nutrient component are based on half-strength Murashige-Skoog (1/2 MS) medium^22^ with sucrose (please see Supplementary Table 1 for details). To make the solid media with containing part of nutrient components, some of these nutrients were added to nutrient-free water and then solidified, unless concentrations were specified otherwise. Note that since the ionic concentration affects the hardness of INA agar, we varied INA agar concentrations of the media to match the hardness approximately. For chemical treatments, a chemical stock solution or DMSO was added to each solid medium just before seed sowing with a final DMSO concentration of 0.1% (v/v). Plate-sown seeds were stratified at 4°C overnight in the dark, then grown at 25°C horizontally in the dark. For host-induced germination assays, seeds were sterilized, sown on solid media without nutrients, and stratified as described above, then placed at a 1-mm distance from the rice roots. The humidity was set at 70% to minimize moisture-derived deviation of the results. Germination percentages, defined by radicle emergence, were periodically scored. For extraction of total RNA, *P. japonicum* seeds were imbibed with nutrient-free water and incubated at 4°C overnight in the dark to break dormancy, flash-frozen in liquid nitrogen, and then homogenized to a fine powder. *Oryza sativa* (japonica, c.v. Shiokari) seeds of wild-type (WT) and *d10*^9^ were unhulled, sterilized for 3 minutes with 70% (v/v) ethanol, and rinsed 5 times with tap water. The, seeds were further sterilized for 20 minutes with a 1:2 (v/v)-diluted commercial bleach solution (Kao, Tokyo, Japan), and rinsed at least 5 times with sterilized water. The surface-sterilized seeds were sown on solid media without nutrients and grown vertically for 4–6 d in short-day conditions (12-h light (~130 μmol m^−2^s^−1^) at 28°C/12-h dark at 25°C). Seedlings were transferred to new solid media without nutrients. After placing *P. japonicum* seeds, plates were turned upside down and incubated in the dark at 25°C.

### Total RNA extraction and RT-qPCR

Total RNA samples from *P. japonicum* seeds were extracted using a Maxwell RSC Plant RNA Kit (Promega, Cat. No. AS1500) and a Maxwell RSC 48 instrument (Promega, Cat. No. AS8500). Reverse transcription and RT-qPCR were performed as previously described.^23^ *Polyubiquitin C 2* (*UBC2*) was used as a reference gene to normalize expression levels. Primers used for RT-qPCR in this study are listed in Supplementary Table 2.

### Data availability

Gene IDs and accession numbers of the *P. japonicum* genes investigated in this study are provided in Supplementary Table 3.

## Results and discussion

### Germination of *P.* *japonicum* is dependent on ambient levels of nitrate ions

**Figure 1.**
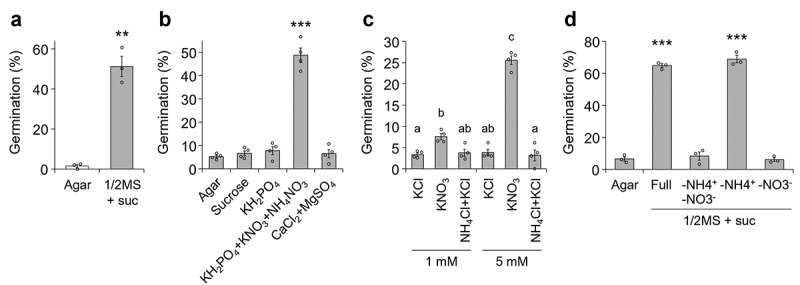
**Germination of *P. japonicum* under various nutritional conditions. a-d**, percentage of germinated seedlings. seedlings were sown on: (**a**) agar without nutrients or 1/2 MS + suc; (**b**) agar containing varying MS macronutrients or sucrose; (**c**) agar containing reduced nitrogen sources; and (d) 1/2 MS + suc agar with limited nitrogen source(s). germination percentages were calculated 3 (**a-c**) or 5 (**d**) d after incubation at 25°C in the dark. representative data are shown (3 or 4 independent batches, 27–106 seeds per batch). data represent means ± SEM. **a,b,d**, **P < 0.01, ***P < 0.001 (Welch’s *t*-test), in comparison with the germination percentage on agar without nutrients. **c**, different letters indicate a statistical significance at *P* < 0.05 (two‐way ANOVA, Tukey’s multiple comparison test).

Since seed germination is often enhanced by light-induced biosynthesis of the phytohormone gibberellin,^24^ we germinated *P. japonicum* seeds in dark conditions to exclude the effects of light. The germination percentage on agar without any supplementary nutrients was lower than that on the 1/2 MS agar with sucrose,^22^ a nutrient-rich medium that has been used for *P. japonicum* germination^17,18,23^ (Figure 1a). We noticed that the germination percentage of *P. japonicum* seeds in the dark was only around 50% even on 1/2 MS with sucrose, in contrast to around 100% germination of *A. thaliana* seeds in the dark.^17^ This difference suggests that the lower germination percentage might reflect the nature of *P. japonicum* seeds. To identify the nutrient component(s) that enhance germination, we measured germination percentages on water agar containing each of the MS macronutrients or sucrose. As we previously observed, adding only nitrogen sources including nitrate ions (NO_3_^−^) and ammonium ions (NH_4_^+^) (KNO_3_ and NH_4_NO_3_) to the media was toxic to *P. japonicum*.^17^ Thus, we also added KH_2_PO_4_ with the nitrogen sources to the test media. We found that nitrate ions significantly promoted germination (Figure 1b,c). Removal of nitrate ions from 1/2 MS with sucrose impaired the germination percentages to a level comparable to agar without nutrients, demonstrating that nitrate ions, but not other nutrient components, including sucrose, contribute to the germination of *P. japonicum* (Figure 1d). Consistent with the germination-stimulating abilities of nitrate ions in non-parasitic plants,^25,26^ our findings suggested that *P. japonicum*, and potentially facultative hemiparasitic plants in the family Orobanchaceae, can germinate by sensing the presence of nitrate ions. It was surprising that phosphate did not affect germination of *P. japonicum*, due to discrepancies in previous findings: biosynthesis and release of SLs are induced in plants in phosphate-deplete conditions, which induces symbiosis with arbuscular mycorrhizal fungi and germination of seeds of neighboring obligate parasitic plants in the family Orobanchaceae.^27^ We hypothesize that this discrepancy might indicate that *P. japonicum* deprives nitrate ions rather than phosphate. Our hypothesis is supported by the previous study showing that *P. japonicum* efficiently deprive nitrogen of host plants, particularly in nutrient-deplete conditions.^28^

### Exogenous strigolactone stimulates germination of *P.* *japonicum* in nitrate-deficient conditions

**Figure 2.**
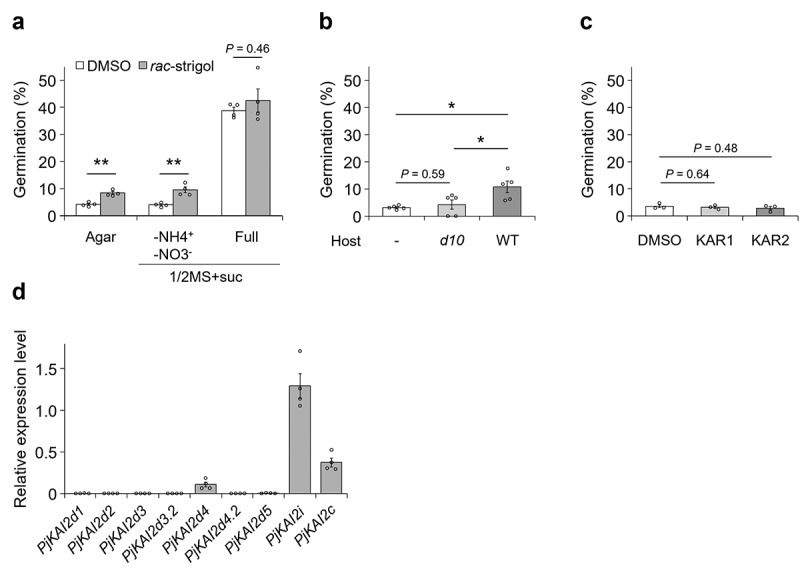
**Effects of exogenous butenolide compounds on germination of *P. japonicum*. a-c**, percentage of germinated seedlings. (**a**) seeds treated with 1 µM *rac*-strigol on agar without nutrients, 1/2 MS + suc without nitrogen sources, or 1/2 MS + suc, (**b**) seeds placed at a 1-mm distance from the rice roots on agar without nutrients, (**c**) seeds treated with 10 µM karrikin 1 (KAR1) or karrikin 2 (KAR2) on agar without nutrients. germination percentages were calculated 3 d after incubation at 25°C in the dark. Representative data are shown (3–5 independent batches, 12–150 seeds per batch). *P < 0.05, **P < 0.01 (Welch’s *t*-test). **d**, Relative expression levels of *PjKAI2* genes in dormancy-breaking seeds. representative data are shown using *pjubc2* as the reference gene (4 technical replicates). data represent the mean ± SEM. experiments were performed three times with similar results. **a,c**, DMSO (0.1% (v/v)) served as the control.

Next, we germinated *P. japonicum* in the presence of germination-stimulant candidates. Under nitrogen-deficient conditions, regardless of other nutrient components, *rac*-strigol promoted germination, a result consistent with the SL-stimulated germination of obligate parasites^6,11^ (agar or 1/2 MS agar with sucrose -NH_4_^+^ -NO_3_^−^ in Figure 2a). By contrast, *rac*-strigol did not significantly affect germination on 1/2 MS agar with sucrose. Note that these germination percentages are much lower than those on 1/2 MS agar with sucrose, suggesting that SLs only partially substitute for the germination-stimulating ability of nitrate. Importantly, the increase of the germination rate was detected when seeds were placed close to WT rice root, but not to SL-deficient *d10* root (Figure 2b). Unlike *rac*-strigol, KARs did not enhance germination in *P. japonicum* even at a higher concentration (Figure 2c). These data suggest that germination in *P. japonicum* in response to stimulants in nutrient-deficient conditions appears to be specific to SLs, a finding consistent with potential SL-specific chemotropic responses in nutrient-poor conditions.^17^ Since chemotropism to SLs is activated via KAI2d^17^, it is possible that KAI2d also positively regulates SL-promoted germination in *P. japonicum* in the absence of nitrate ions. We found that *PjKAI2i, PjKAI2c*, and *PjKAI2d4* were expressed in seeds whose dormancy was broken in nutrient-free water, with *PjKAI2i* having the highest expression (Figure 2d). As shown in the previous studies, KAI2i and KAI2c might recognize KARs and/or KLs rather than SL,^11,14^ in contrast to KAI2d recognizing SLs but not KARs.^11,16^ Therefore, our data suggest a possibility that the seeds are ready to recognize germination-stimulating signals via either endogenous KLs, probably by PjKAI2i or PjKAI2c, or exogenous SLs, likely by PjKAI2d4 (Figure 2a,d). Among the *PjKAI2d* homologs, expression of *PjKAI2d4* was the highest, whereas expression of the other six *PjKAI2d* genes, including *PjKAI2d2* and *PjKAI2d3.2*, which contribute to root chemotropism to SLs, was much lower (Figure 2d). This result indicates that different KAI2d genes are likely activated to recognize exogenous SLs during germination and chemotropism.

**Figure 3.**
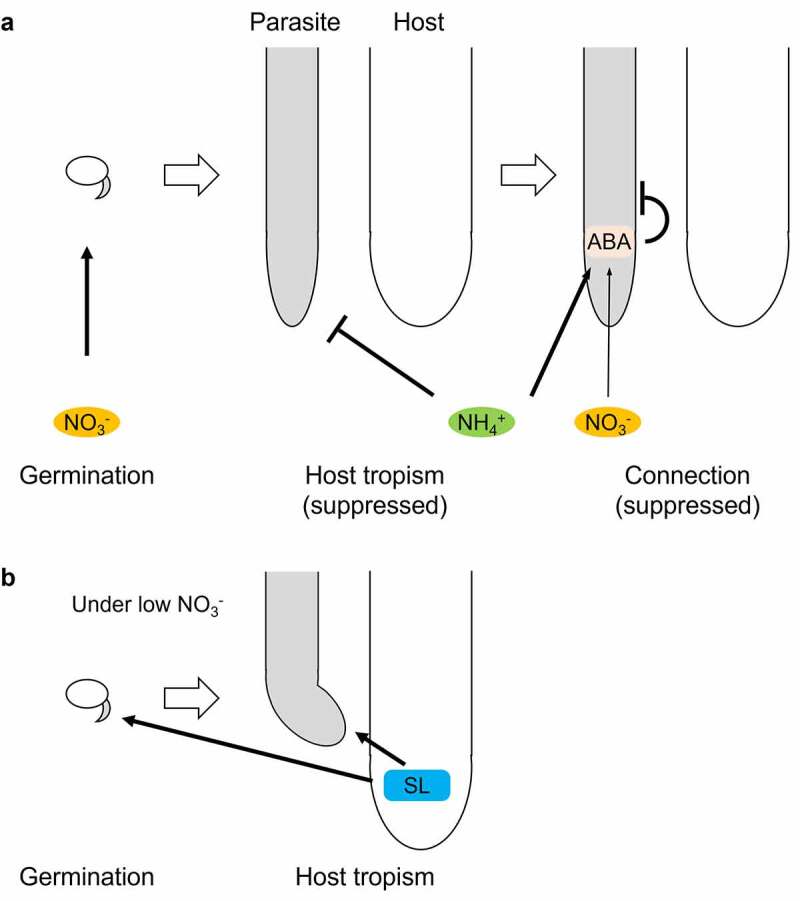
**A schematic model of host-invading processes in *P. japonicum***. Effects of nitrogen sources, abscisic acid (ABA)^29^ (a), and strigolactone (SL) (b) on germination, host tropism,^17^ and connection.^29^ black arrows and T bars depict positive and negative regulation, respectively. thick and thin arrows represent strong and weak contributions, respectively.

We found that *P. japonicum* germination was suppressed in the absence of nitrate ions. In such conditions, *P. japonicum* may be able to germinate by recognizing host-derived SLs. This strategy might be unique to facultative parasites; germination proceeds only in the presence of nitrogen in the soil or hosts that can provide nitrogen once the infection is established. Interestingly, tropism to hosts and infection of hosts are both inhibited in the presence of nitrogen sources.^17,29^ Thus, when nitrogen is rich enough in the soil, *P. japonicum* is likely to germinate well and avoid parasitism (Figure 3a). SLs can be the assurance of host presence and become important only when nitrogen cannot be acquired from the soil (Figure 3b). A recent study showed that nitrogen impairs an early stage in the formation of an invasive organ called a haustorium and that ammonium ions have stronger inhibitory activity than nitrate ions. Nitrogen-blocked haustorium formation is accomplished through the accumulation of abscisic acid (ABA), a phytohormone that affects haustorium formation.^29^ These findings support the hypothesis that *P. japonicum* uses SLs for germination and host tropism only in nitrogen-poor conditions, which are optimal for haustorium formation (Figure 3a,b). Why nitrate ions are important for germination, whereas ammonium ions inhibit the tropism, is unclear and will be a topic of future studies. Perhaps, the mechanisms for nitrogen absorption may differ in seeds and roots. We also found that the expression of *PjKAI2i* was higher than *PjKAI2d* and *PjKAI2c* (Figure 2d). KAI2i in Lamiales have been converted from KAI2 in angiosperms, including KAI2c, and are phylogenetically separate from KAI2d.^11,^^17^ Despite their phylogenetic uniqueness, the functions of KAI2i, including PjKAI2i, remain unknown. Future studies on KAI2i should help reveal how *P. japonicum* germination is stimulated by signals via PjKAI2i.

In conclusion, our study has revealed a unique germination control mechanism in *P. japonicum*. Since experimental systems, including genetic tools to elucidate the molecular mechanism underlying parasitism, have been established in *P. japonicum*,^17,18,23,30,31^ we expect further development of genetic tools, such as a stable and heritable transformation system, will aid in elucidating the mechanism for choosing independent living versus parasitism. Future findings might provide a means for controlling infestations of major cereals by Orobanchaceae parasitic plants.

## Supplementary Material

Supplemental Material
